# Chinese medicine for COVID-19: A protocol for systematic review and meta-analysis

**DOI:** 10.1097/MD.0000000000020660

**Published:** 2020-06-19

**Authors:** Huizhen Chen, Ziyan Xie, Yuxia Zhu, Qiu Chen, Chunguang Xie

**Affiliations:** a Chengdu University of Traditional Chinese Medicine; b Hospital of Chengdu University of Traditional Chinese Medicine, Jinniu District, Chengdu, China.

**Keywords:** Chinese medicine, coronavirus disease 2019, protocol, systematic review

## Abstract

**Background:**

Coronavirus disease 2019 (COVID-19) is a respiratory illness that can spread from person to person. The virus that causes COVID-19 is a novel coronavirus that was first identified during an investigation into an outbreak in Wuhan, China. The clinical spectrum of SARS-CoV-2 infection appears to be wide, encompassing asymptomatic infection, mild upper respiratory tract illness, and severe viral pneumonia with respiratory failure and even death, with many patients being hospitalised with pneumonia. In China and East Asia, Chinese medicine has been widely used to treat diverse diseases for thousands of years. As an important means of treatment now, Chinese medicine plays a significant role in the treatment of respiratory diseases in China. The aim of this study is to assess the efficacy and safety of Chinese medicine for COVID-19.

**Methods:**

We will search the following sources for the identification of trials: The Cochrane Library, PubMed, EMBASE, Chinese Biomedical Literature Database (CBM), Chinese National Knowledge Infrastructure Database (CNKI), Chinese Science and Technique Journals Database (VIP), and the Wanfang Database. All the above databases will be searched from the available date of inception until the latest issue. No language or publication restriction will be used. Randomized controlled trials will be included if they recruited participants with COVID-19 for assessing the effect of Chinese medicine vs control (placebo, no treatment, and other therapeutic agents). Primary outcomes will include chest CT and nucleic acid detection of respiratory samples. Two authors will independently scan the articles searched, extract the data from articles included, and assess the risk of bias by Cochrane tool of risk of bias. Disagreements will be resolved by consensus or the involvement of a third party. All analysis will be performed based on the Cochrane Handbook for Systematic Reviews of Interventions. Dichotomous variables will be reported as risk ratio or odds ratio with 95% confidence intervals (CIs) and continuous variables will be summarized as mean difference or standard mean difference with 95% CIs.

**Results and Conclusion:**

The available evidence of the treatment of COVID-19 with traditional Chinese medicine will be summarized, and evaluation of the efficacy and the adverse effects of these treatments will be made. This review will be disseminated in print by peer-review.

## 1 Introduction

In December 2019, Wuhan, the capital of Hubei Province, China, became the center of the outbreak of pneumonia of unknown causes. By January 7, 2020, Chinese scientists had isolated a new coronavirus, severe acute respiratory syndrome coronavirus type 2 (SARS-coV-2),^[1]^ which was named as coronavirus disease 2019 (COVID-19) by WHO in February 2020.^[2]^ Despite the outbreak is likely to start with a zoonotic epidemic related to a large seafood market, which also trades live wildlife, it soon became clear that effective human to human transmission is also taking place.^[3]^ New major epidemic foci of COVID-19, some of which have no traceable origin, are expanding rapidly in Europe, Asia, North America, and the Middle East.^[4]^ The clinical scope of SARS-CoV-2 infection seems to be very wide, including asymptomatic infection, mild upper respiratory disease, severe viral pneumonia with respiratory failure, or even death. Many patients are hospitalized for pneumonia.^[5–7]^ Human is a complete mixture, therefore, COVID-19 is also related to many factors, including its internal and external causes, such as age, gender, anamnesis, and so on. So far, COVID-19 vaccine has not been successfully developed. At present, the treatment of SARS-CoV-2 infection is mainly symptomatic. Hence, empiric antibiotics, antiviral therapy (oseltamivir), and systemic corticosteroids are often used for treatment. Invasive mechanical ventilation was performed in patients with refractory hypoxemia. Holshue et al^[8]^ used remdesivir to treat SARS-CoV-2 infection and achieved good results. Lu^[9]^ speculated that in addition to antiviral agents and antibiotics, neuraminidase inhibitors, RNA synthesis inhibitors, and Chinese medicine could also be used to treat COVID-19. And according to every version of diagnosis and treatment plan of COVID-19 of China, Chinese medicine is a vital part.

Chinese medicine is extracted from plant sources to treat diseases. In China and East Asia, for thousands of years, it has been widely used to treat various diseases. In western countries, Chinese medicine as a form of complementary medicine is increasingly accepted.^[10]^ Researches shows that the combination of Chinese medicine and western medicine can improve the clinical symptoms and quality of life better than the western medicine alone.^[11]^ For decades, Chinese medicine has also participated in the intervention of respiratory diseases, greatly enriching the treatment of respiratory diseases, including viral pneumonia.^[11–18]^

Recently, Chinese medicine has demonstrated a good therapeutic effect on COVID-19. However, based on the application of Western medicine, the efficacy of traditional Chinese medicine on COVID-19 still remains to be verified. Therefore, our purpose is to collect the latest information of COVID-19 on Chinese medicine, evaluate the therapeutic effect of Chinese medicine, and provide help for clinical decision-making.

## 2 Methods and analysis

### 2.1 Objectives and registration

This article will evaluate the efficacy and safety of Chinese medicine on COVID-19. This review protocol has been registered in the International Prospective Register of Systematic Reviews (PROSPERO: CRD42020175105). In addition, this review will follow the Preferred Reporting Items for Systematic Reviews and Meta-Analyses Statement (PRISMA-P reporting guidelines).^[19]^

Review question:

How does therapeutic effects of a traditional Chinese medicine plus western medicine impact on COVID-19 compared to western medicine alone?

### 2.2 Eligibility criteria

#### 2.2.1 Types of studies

Randomized controlled trials (RCTs) will be included in this system review, regardless of publication status and language.

#### 2.2.2 Types of participants

Participants with COVID-19 will be included regardless of their age, sex, or race.

#### 2.2.3 Patient and public involvement

In this study, there is no patient and public involvement in consideration of this protocol for a systematic review.

#### 2.2.4 Types of interventions

All types of Chinese medicine will be included. There is no limitation on the number of herbs, administration methods, dosage, or duration of treatment. The comparisons will be either with other therapeutic agents, or with no other treatment or placebo based on conventional treatment of western medicine.

### 2.3 Types of outcome measures

Primary outcomes: chest CT, nucleic acid detection of respiratory samples.

Secondary outcomes: symptoms (fever, cough, shortness of breath, diarrhea, etc)

### 2.4 Search methods

#### 2.4.1 Electronic searches

We will search the following sources for the identification of trials: the Cochrane Library, PubMed, EMBASE, Chinese Biomedical Literature Database, Chinese National Knowledge Infrastructure Database, Chinese Science and Technique Journals Database, and the Wanfang Database. All the above databases will be searched from the available date of inception until the latest issue (March 2020). No language or publication restriction will be used.

#### 2.4.2 Other sources

We will scan the reference lists of reviews and retrieve articles for additional studies.

#### 2.4.3 Search strategy

Search terms (free words search) are as follows: the search terms used are (traditional Chinese medicine or traditional medicine, Chinese or herbal medicine or herbs or Chinese medicine) and (2019 novel coronavirus infection or COVID19 or COVID-19 or coronavirus disease 2019 or coronavirus disease-19 or 2019-nCoV disease or 2019 novel coronavirus disease or 2019-nCoV infection) and (randomized clinical trial or randomized or RCT).

### 2.5 Data collection and analysis

#### 2.5.1 Selection of studies

In order to identify studies, the 2 review authors (HC and ZX) will independently scan the titles and abstracts of all articles identified from the electronic database. All potentially relevant articles will be scanned. If there is any disagreement to the selection of the article, it will be discussed with the third (QC) author. The selection process will be shown in the Preferred Reporting Items for Systematic Review and Meta-analysis flow chart.

#### 2.5.2 Data extraction and management

For all studies included, 2 reviewers (HC and YZ) will extract relevant information independently. Information will include year of publication, author, participant, intervention, control, duration of intervention, outcome, and methodological characteristics. The disagreement will be settled by the another reviewer (QC).

#### 2.5.3 Assessment of the risk of bias in the included studies

Risk of bias will be independently assessed by 2 authors (HC and ZX) using the Cochrane tool of risk of bias. The following items will be evaluated: assignment hiding (selection bias), incomplete result data (loss bias), selection result report (reporting bias), and other biases. Studies will be evaluated high, low and unclear. The disagreement will be settled by another reviewer (QC).

#### 2.5.4 Measurement of the treatment effect

Continuous variables will be reported as mean difference with 95% confidence intervals (CIs). For different measurement scales, standardized mean difference analysis with 95% CI will be used. Categorical variables will be summarized as risk ratios or odds ratio with 95% CIs. All analysis will be performed based on the continuous variables will be reported as the mean difference of the 95% CIs. Standardized mean difference analysis with 95% confidence interval will be used for different measurement scales. The categorical variables will be summarized as risk ratio or odds ratio and 95% CIs. All analyses will be conducted in accordance with the Cochrane Handbook for Systematic Reviews of Interventions.^[20]^

#### 2.5.5 Units of analysis issues

All studies of parallel design will be included in this review. For cross-over trials, only data from the first treatment period were analyzed. For studies with multiple control groups, the unit of analysis will be used for each of the control groups.

#### 2.5.6 Dealing with missing data

If the information is insufficient or missing, we will contact the study author. If we fail to obtain sufficient data, we will assume dichotomous outcomes for patients not experiencing any change in their clinical outcome variables. Sensitivity analyses will be performed to assess how sensitive the results are to changes in the assumptions made.

#### 2.5.7 Assessment of heterogeneity

Heterogeneity will be identified by visual inspection of the forest and tested by standard Chi-squared statistic and a significance level of 0.1. In addition, the I^2^ statistic will be used to test heterogeneity to quantify inconsistency. Fixed or random effects models will be performed in meta-analysis. If I^2^ >0.5, the random effects models will be used.^[20]^

#### 2.5.8 Assessment of reporting biases

We will use funnel plots to assess the potential for small study bias if there are enough studies. The asymmetry of funnel plots will show the possible small research effects.^[21,22]^

#### 2.5.9 Data synthesis

If there are sufficient studies and comparable outcomes, we will perform a meta-analysis using random effect modeling.

Chinese medicine plus western medicine versus Western medicine
Chinese medicine plus western medicine versus placebo plus Western medicine

#### 2.5.10 Subgroup analysis

Subgroup analysis will be performed to explore the differences in the methodologic quality, gender, age, race/ethnicity, and types of Chinese medicine.

#### 2.5.11 Sensitivity analysis

Sensitivity analysis will be performed to test the robustness of findings if there are sufficient studies included. The factors on effect are as follows:

methodologic quality: analysis will be performed excluding studies of poor methodologic quality
sample size: analysis will be performed excluding small sample size studies
diagnostic criteria: analysis will be performed in studies of the same diagnostic criteria

#### 2.5.12 Confidence in cumulative evidence

The evidence level of the results will be assessed using a methodology based on the Grading of Recommendations Assessment, Development and Evaluation. The quality of the evidence will be assessed based on several factors including research limitations, effect consistency, imprecision, indirectness, and publication bias. The assessments will be categorized as high quality, medium quality, low quality and very low quality.

## 3 Discussion

The effective treatment of COVID-19 is of great significance. Chinese medicine could help make up for the deficiency of current treatment of COVID-19,^[23]^ which is worth studying. We will summarize the available evidence of the treatment of COVID-19 with traditional Chinese medicine, and evaluate the efficacy and the adverse effects of these treatments. Our findings may help clinicians and health professionals to make clinical decisions about the treatment of patients with COVID-19.

### 3.1 Strengths and limitations of this study

- Study selection, data extraction, and quality assessment will be performed independently by 2 researchers, which will ensure that all relevant studies are included without personal biases.
- This systematic review and meta-analysis will be the first addressing Chinese medicine for COVID-19 and be the foremost evidence for this potentially fatal disease.
- This study will focus on relevant clinical information used in clinical practice, which may facilitate the application of the review's findings to the clinical setting.
- There may be high heterogeneity from the various evaluation standards in different prescription of Chinese medicine.

### 3.2 Ethics and dissemination

Ethical approval is not required as this protocol is for a systematic review. In this study, participants are not recruited and data are not collected from participants. The review will be disseminated through peer-reviewed publications.

## Author contributions

**Conceptualization:** Huizhen Chen, Chunguang Xie.

**Data curation:** Huizhen Chen, Ziyan Xie.

**Formal analysis:** Huizhen Chen, Yuxia Zhu.

**Methodology:** Huizhen Chen, Qiu Chen.

**Project administration:** Huizhen Chen.

**Software:** Huizhen Chen.

**Supervision:** Chunguang Xie.

**Writing – original draft:** Huizhen Chen.

**Writing – review & editing:** Huizhen Chen, Qiu Chen, Chunguang Xie.
